# Adoptive therapy with amyloid-β specific regulatory T cells alleviates Alzheimer's disease

**DOI:** 10.7150/thno.75965

**Published:** 2022-11-07

**Authors:** HyeJin Yang, Seon-Young Park, Hyunjung Baek, Chanju Lee, Geehoon Chung, Xiao Liu, Ji Hwan Lee, Byungkyu Kim, Minjin Kwon, Hyojung Choi, Hyung Joon Kim, Jae Yoon Kim, Younsub Kim, Ye-Seul Lee, Gaheon Lee, Sun Kwang Kim, Jin Su Kim, Young-Tae Chang, Woo Sang Jung, Kyung Hwa Kim, Hyunsu Bae

**Affiliations:** 1 Department of Physiology, College of Korean Medicine, Kyung Hee University, 26-6 Kyungheedae-ro, Dongdaemoon-gu, Seoul 02453, Korea; 2 Cancer Immunology Branch, National Cancer Center, 323 Ilsan-ro, Ilsandong-gu, Goyang 10408, Korea; 3 Department of Chemistry, Pohang University of Science and Technology, Pohang 37673, Korea; 4 Institute of Life Science & Biotechnology, VT Bio. Co., Ltd. 3 rd FL, 16 Samseong-ro 76-gil, Gangnam-gu, Seoul 06185, Korea; 5 Department of Anatomy and Acupoint, College of Korean Medicine, Gachon University, Seongnam 13120, Korea; 6 Department of Health Sciences, The Graduate School of Dong-A University, 840 Hadan-dong, Saha-gu, Busan 49315, Korea; 7 Division of RI Application, Korea Institute Radiological and Medical Sciences, 75 Nowon-ro, Nowon-Gu, Seoul 01812, Korea; 8 Center for Self-assembly and Complexity, Institute for Basic Science (IBS), Pohang 37673, Korea; 9 Stroke center, Kyung Hee University, 26-6 Kyungheedae-ro, Dongdaemoon-gu, Seoul 02453, Korea

**Keywords:** Neuroinflammation, antigen-specific Tregs, adoptive transfer, microglia, bystander suppression

## Abstract

**Rationale**: Neuroinflammation is a primary feature of Alzheimer's disease (AD), for which an increasing number of drugs have been specifically developed. The present study aimed to define the therapeutic impact of a specific subpopulation of T cells that can suppress excessive inflammation in various immune and inflammatory disorders, namely, CD4^+^CD25^+^Foxp3^+^ regulatory T cells (Tregs).

**Methods**: To generate Aβ antigen-specific Tregs (Aβ^+^ Tregs), Aβ 1-42 peptide was applied *in vivo* and subsequent *in vitro* splenocyte culture. After isolating Tregs by magnetic bead based purification method, Aβ^+^ Tregs were adoptively transferred into 3xTg-AD mice via tail vein injection. Therapeutic efficacy was confirmed with behavior test, Western blot, quantitative real-time PCR (qRT-PCR), enzyme-linked immunosorbent assay (ELISA), and immunohistochemistry staining (IHC). *In vitro* suppression assay was performed to evaluate the suppressive activity of Aβ^+^ Tregs using flow cytometry. Thy1.1^+^ Treg trafficking and distribution was analyzed to explore the infused Tregs migration into specific organs in an antigen-driven manner in AD mice. We further assessed cerebral glucose metabolism using ^18^F-FDG-PET, an imaging approach for AD biological definition. Subsequently, we evaluated the migration of Aβ^+^ Tregs toward Aβ activated microglia using live cell imaging, chemotaxis, antibody blocking and migration assay.

**Results**: We showed that Aβ-stimulated Tregs inhibited microglial proinflammatory activity and modulated the microglial phenotype via bystander suppression. Single adoptive transfer of Aβ^+^ Tregs was enough to induce amelioration of cognitive impairments, Aβ accumulation, hyper-phosphorylation of tau, and neuroinflammation during AD pathology. Moreover, Aβ-specific Tregs effectively inhibited inflammation in primary microglia induced by Aβ exposure. It may indicate bystander suppression in which Aβ-specific Tregs promote immune tolerance by secreting cytokines to modulate immune responses during neurodegeneration.

**Conclusions**: The administration of Aβ antigen-specific regulatory T cells may represent a new cellular therapeutic strategy for AD that acts by modulating the inflammatory status in AD.

## Introduction

Recent advances in immunotherapies have generated new powerful approaches for targeting neurological disorders. Specifically, regulatory T cell therapy is reshaping the landscape of brain-related disorder treatment. Regulatory T cells (Tregs) are a small subset of T cells that modulate the immune system and maintain immune homeostasis 1, 2. Many studies performed with patients and animal models of ischemic stroke provide evidence that Tregs can block hemorrhage and improve sensorimotor functions 3. Interestingly, when Tregs were intravenously administered to ischemic mice, hemorrhagic transformation in the brain was dramatically alleviated, suggesting a neuroprotective effect mediated by the infused Tregs. Beyond their effects on autoimmune brain disorders, the therapeutic impact of Tregs is now being translated to the treatment of neurodegenerative diseases. Despite the general consensus on neuroinflammation as a therapeutic target in AD, it remains unclear whether the adoptive transfer of Tregs is beneficial for the AD brain.

Our group previously developed a novel amyloid-beta (Aβ) vaccination strategy in an AD mouse model 4. When considering immune-based therapeutic effects, regulatory T cells controlled neuroinflammation during neurodegeneration in AD model mice. As Treg induction itself can induce improved disease outcomes during neurodegeneration 5. We investigated a novel methodology eliciting biological activities similar to those achieved with novel immune-related therapies. Here, we presented a strategy to generate Aβ-specific Tregs with both *in vivo* and *in vitro* stimulation against Aβ. Compared to polyclonal PBS-stimulated Tregs, Aβ^+^ Tregs displayed robust and stable immunosuppressive phenotypes. Surprisingly, adoptive transfer of Aβ^+^ Tregs induced therapeutic benefits against AD including improving cognitive impairments and decreasing Aβ accumulation in animal model of AD. The functional suppression of Aβ^+^ Tregs against AD seemed to modulate neuroinflammation, by mainly controlling microglial activation. Altogether, our findings suggest that Treg immunotherapy may represent a new therapeutic strategy for treating AD.

## Materials and Methods

### Animals

Six- to seven-week male six mice depleted of Tregs (DEREG, C57BL/6^DTR^/^eGFP^) (The Jackson Laboratory), which were engineered to express diphtheria toxin (DT) receptor with green fluorescent protein (GFP) under the control of *Foxp3*, were used to evaluate the characteristics of Tregs. For *in vivo* Treg trafficking experiments, three to five Thy1.1 (B6. PL-Thy1<a>/CyJ) mice were used for the production of antigen-specific Tregs. Transgenic 3xTg-AD (B6;129-Tg (APPSwe, tauP301L)1Lfa *Psen1^tm1Mpm^*/Mmjax) mice (The Jackson Laboratory) were used as the recipients (five to six per group) of Aβ^+^ Tregs. Wild-type (WT) littermate (B6;129SF2/J) mice were used as non-transgenic (non-Tg) controls. All animal studies were ethically reviewed and performed in compliance with the ARRIVE guidelines 6. Animal experiments were randomly divided into each group and conducted according to the rules for animal care and the guiding principles for experiments using animals and were approved by the University of Kyung Hee Animal Care and Use Committee (KHUASP(SE)-18-002, KHUASP(SE)-20-240, and KHUASP(SE)-21-102).

### Depletion of regulatory T cells and *in vivo* production of Aβ antigen-specific Tregs

DEREG mice were treated with DT (Sigma-Aldrich) to deplete pre-existing Tregs. DT (1 µg) in 100 µl of PBS was administered intraperitoneally (i.p.) to each mouse on days -3 and -1 before Aβ injection. For Aβ preparation, the human Aβ 1-42 peptide (GenScript) was used to create a stock solution at a concentration of 2 mg/mL in a sterile saline solution, followed by incubation at 37 °C overnight for aggregation. An aggregated Aβ and complete Freund's adjuvant (CFA; Sigma-Aldrich) mixture (1:1) was administered subcutaneously (s.c.) on Day 0 (100 µg/mouse). To generate antigen-specific Tregs with Aβ (Aβ^+^ Tregs), mice were additionally administered aggregated Aβ. Keyhole limpet hemocyanin (KLH; Sigma-Aldrich) was administered subcutaneously (s.c.) to generate KHL-treated Tregs (KLH^+^ Tregs). Neither KLH nor Aβ was used to produce polyclonal Tregs (PBS^+^ Tregs). On Days 1 and 5, 0.5 mg/kg bvPLA2 (Sigma-Aldrich) was administered i.p. when generating Aβ^+^ Tregs and KLH^+^ Tregs. For PBS^+^ Treg generation, only PBS was administered to the mice, not bvPLA2. On Day 10, the mice were euthanized, and the spleen was removed to generate an *in vitro* culture of Treg cells.

### *In vitro* culture of murine Tregs

Splenocytes from DEREG mice used to generate PBS^+^ Tregs, Aβ^+^ Tregs or KLH^+^ Tregs were obtained by mechanical disruption of spleen. The splenocytes were passed through a 40-μm cell strainer and cultured with 5 µg/mL plate-bound anti-CD3ε antibodies in combination with 2 µg/mL soluble anti-CD28 antibodies (BD Biosciences). The cells were stimulated with Aβ (10 µg/mL) or KLH (10 µg/mL) and bvPLA2 (0.4 µg/mL). After 4 days, CD4^+^CD25^+^ Tregs were isolated using magnetic-activated cell sorting (MACS) according to the manufacturer's protocol (CD4^+^CD25^+^ Regulatory T Cell Isolation Kit, Miltenyi Biotec).

### Adoptive transfer of mouse Tregs

PBS^+^, Aβ^+^ or KLH^+^ Tregs (1 × 10^6^) from DEREG mice were adoptively transferred into 4-month-old 3xTg-AD mice via tail vein injection. To eliminate the transferred Tregs, the recipient mice were injected with DT 1 day, 1 week, or 1 month after the Treg transfer. The mice were assessed with behavior tests and sacrificed at the indicated time points.

### *In vitro* suppression assay

Aβ-specific CD4^+^CD25^-^ effector T cells (Aβ-Teffs) were isolated from the splenocytes of Aβ-immunized DEREG mice using a CD4^+^CD25^+^ Regulatory T Cell Isolation Kit (Miltenyi Biotec) and labeled with Cell Proliferation Dye eFluor 670 (e670; Invitrogen, #65-0840-85) according to the manufacturer's protocol. Labeled Aβ-Teffs were seeded at 1 × 10^5^ cells per well in round-bottom 96-well plates coated with 5 µg/mL anti-CD3e antibodies and containing 2 µg/mL soluble anti-CD28 antibodies and cocultured with Aβ^+^, KLH^+^ or PBS^+^ Tregs at a ratio of 1:1, 1:2 or 1:4 (Treg:Teff). After 3 days, Teff proliferation was analyzed as previously described 7. % Suppression was calculated as 100 - (proliferation [Treg coculture]/proliferation [Teff only]) × 100.

### Thy1.1^+^ Treg distribution

To compare the distributions of PBS^+^, Aβ^+^ and KLH^+^ Tregs, splenocytes from Thy1.1 mice were stimulated with Aβ or KLH as described for the *in vitro* culture of murine Tregs. After 4 days, the Tregs were isolated, and 1 × 10^6^ Tregs were adoptively transferred into 4-month-old 3xTg (Thy 1.2^+^) mice. After 7 days, mice were euthanized and the inguinal lymph nodes, spleen, and blood were harvested. Mice were perfused to harvest the lungs, kidneys, liver, and brain. T cells were enriched from the lungs, kidneys, liver, and brain by using 30-70% Percoll (GE Healthcare, #17-5445-02) density gradient centrifugation and Debris Removal Solution (Miltenyi, #130-109-398).

### ^18^F-FDG Micro-PET scan

^18^F-FDG (1 mCi) was injected via the tail vein, and the injected mice were anesthetized with 2% isoflurane in 100% oxygen. A Siemens Inveon PET scanner (Siemens Medical Solutions, Malvern, PA, USA) was used for PET imaging. The transverse resolution was <1.8 mm at the center. After allowing uptake for 10 min, 30 min of emission PET data were acquired with a 350-650 keV energy window. The list-mode PET data were reconstructed using 3D reprojection methods. The pixel size of the reconstructed images was 0.15 × 0.15 × 0.79 mm^3^. Attenuation, scatter corrections, and normalization were performed. To identify regional differences between groups, a region of interest (ROI) was drawn in the hippocampus. The maximal value of the ROI was calculated within ROI regions to avoid a partial volume effect. ROIs were drawn on at least 10 subsequent coronal sections of PET data, and then the averaged value of the PET counts of individual mice were used for statistics. Asipro Software (Siemens Healthineers, Erlangen, Germany) was used for ROI delineation. For normalization of body weight and injected dose differences, the PET counts of the hippocampus were normalized to the whole-brain counts.

### Coculture of Microglia and Tregs

For primary microglial cultures, brains were removed from C57BL/6 mice on Postnatal Day 1 (The Jackson Laboratory). After removal of the meninges from the cortexes, the brains were treated with 2.5% trypsin and DNase in Hank's balanced salt solution. Mixed glial cells were plated in flasks coated with poly-D-lysine and grown in Dulbecco's Modified Eagle Medium (DMEM) containing 10% FBS and 25 ng/mL Granulocyte-macrophage colony-stimulating factor (GM-CSF; R&D Systems, Minneapolis, MN, USA, #415-ML). Cells were incubated at 37°C with 95% humidity and 5% CO_2_ for all experiments. Microglial cells were harvested by gentle shaking 7-10 days after plating and subjected to coculture with PBS^+^ or Aβ^+^ Tregs. PBS^+^ or Aβ^+^ Tregs were cocultured with primary microglia (microglia:Tregs = 20:1) in 60-mm culture dishes. Within 12 h of coculture, the cells were stimulated with a fibrillated Aβ 1-42 peptide (5 µM, GenScript) for 6 h. A 24-well Transwell insert system (0.4 μm pore size; Corning) was used to prevent direct contact between the microglia (PBS-treated or Aβ-activated microglia for 16 hours) and Tregs (PBS^+^ or Aβ^+^ Tregs). Microglia were plated in serum-free DMEM at 10^5^ cells/well in the bottom chamber, while 5 x 10^3^ cells/well PBS^+^ or Aβ^+^ Tregs were added to the Transwell insert; the cells were cocultured for 6 h. The microglia in the bottom chamber were collected and analyzed in subsequent experiments.

### Live cell imaging

For time-lapse imaging, 5 × 10^4^ primary microglia in 1 mL were seeded in 4-well chambers and cultured for 2-3 h before live cell imaging was performed. Approximately 2.5 × 10^3^ murine PBS^+^ Tregs and Aβ^+^ Tregs were seeded in each chamber that contained microglia. The microglia were continuously observed from preactivation to postactivation with Aβ (5 µM) in the presence of CDr20 (1 µM) every 1 min over a total of 45 min under the red fluorescent channel (excitation: 570 nm, emission: 600 nm). The change in the ROI of each cell was measured for a total of 45 min. All observations were performed using a DeltaVision imaging system (GE Healthcare). To assess fluorescence intensity during live-cell imaging, 50 cells were randomly selected and analyzed using DeltaVision SoftWorX software (v.6.1.3, GE Healthcare). CDr20 was kindly provided by Dr. YT Chang (Pohang University of Science and Technology, Pohang, Korea). The CDr20 stock (1 µl) was dissolved in an appropriate volume of 10 mM dimethyl sulfoxide (Sigma-Aldrich).

### Chemotaxis

PBS^+^ and Aβ^+^ Treg chemotaxis was assayed using 24-well Transwell chambers with 5-μm pores (Corning, Corning, NY). A total of 10^5^ cells in 100 μL of medium (RPMI 1640 medium supplemented with 0.5% BSA) were plated in the upper chambers. KLH or Aβ, diluted in 600 μl of medium, was plated in the lower chambers, and the Transwell system was incubated for 3 h at 37°C. The migrated cells in the bottom wells were collected and counted with a LUNA-II automated cell counter (Logos Biosystems, Anyang, Korea). All experiments were conducted in triplicate.

### Antibody blocking and migration assay

To block the interaction between Aβ-activated microglia and Tregs, we used the following antibodies as blocking antibodies: purified rat IgG (clone RTK2071; BioLegend, #400402), purified hamster anti-mouse CD40 (clone HM40-3; BD Biosciences, #553721), and purified rat anti-CD80 (clone 1G10/B7; BD Biosciences, #553368). The antibodies were diluted in serum-free medium (DMEM, 1:100) and added to Aβ-activated microglia for 16 hours.

To analyze PBS^+^ and Aβ^+^ Treg migration toward activated microglia, we used a cell migration assay kit (Abcam, #ab235691). Briefly, anti-IgG, anti-CD40 or anti-CD80 antibody-treated microglia were plated in serum-free DMEM at 10^5^ cells/well in the bottom chamber of a Transwell system, and 5x10^3^ cells/well PBS^+^ or Aβ^+^ Tregs were plated in the upper chamber and incubated for 6 h. The PBS^+^ or Aβ^+^ Treg cells migrating into the bottom chamber of the Transwell system were counted with a fluorescence plate reader (Fluoroskan Ascent FL, Thermo Scientific, Waltham, MA, USA).

### Statistical analysis

Graphical and statistical analyses were performed using Prism software (GraphPad Software, San Diego, CA, USA). Normally distributed data are expressed as the mean ± standard error of the mean (SEM). Statistical differences in measurements over time were determined using repeated measures analysis of variance (ANOVA) followed by a post-hoc analysis. A *P* value < 0.05 was considered to be statistically significant.

## Results

### Stable Aβ-specific Treg generation by *in vivo* and *in vitro* Aβ immune stimulation

**Figure 1:**
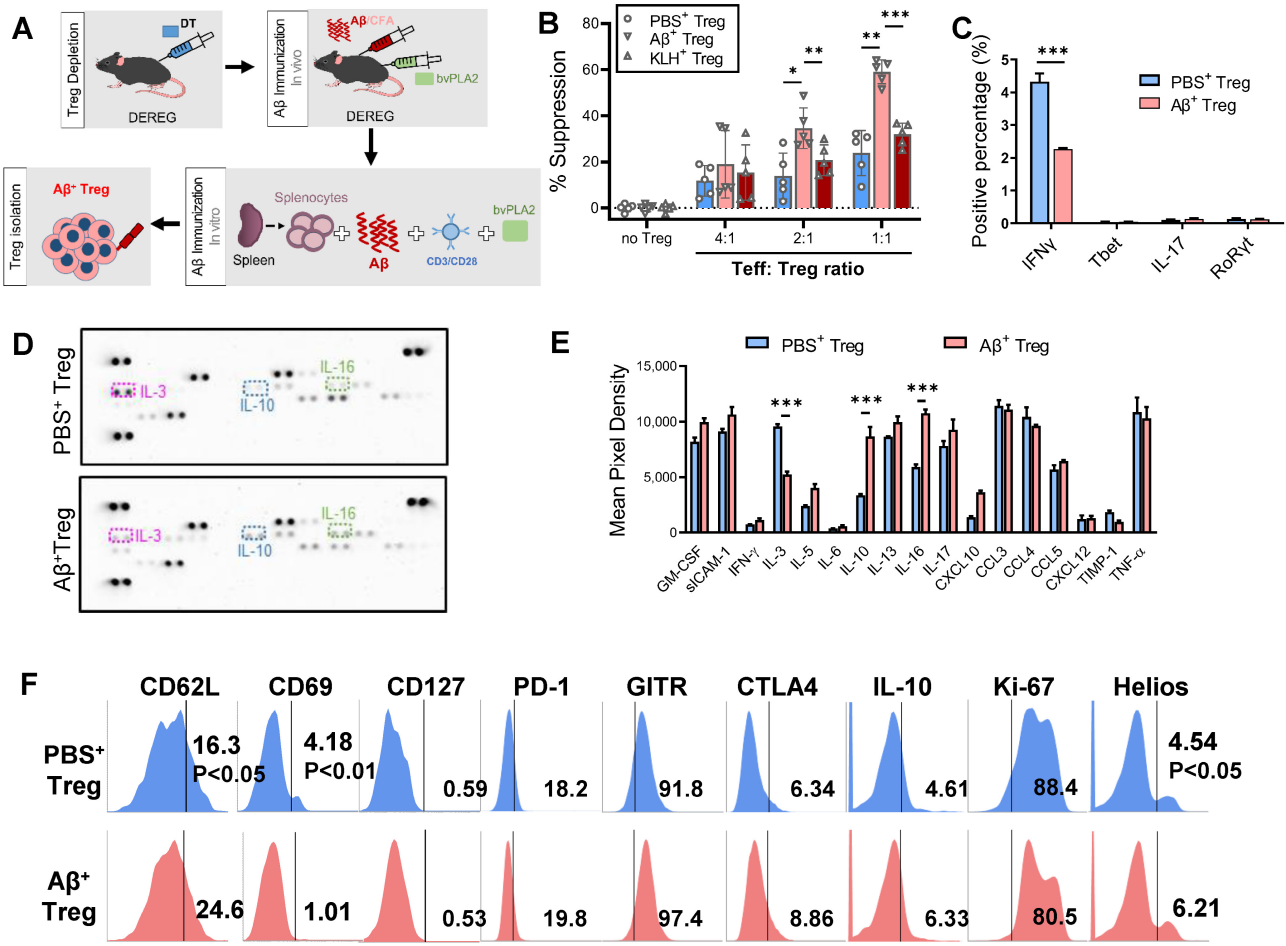
** Stable Aβ-specific Treg generation by *in vivo* and *in vitro* Aβ immune stimulation.** (A) Experimental scheme for the *in vivo* depletion of Tregs and *in vitro* Aβ antigen-specific Treg production and culture. (B) To compare suppressive capacities, PBS^+^, Aβ^+^, and KLH^+^ Tregs were cocultured with e670-labeled Teffs at ratios of 4:1, 2:1 and 1:1 (Teff:Treg). After 3 days, the percent suppression was analyzed. (C) Intracellular cytokine and transcription factor ecpressed by PBS^+^ and Aβ^+^ Tregs were analyzed by flow cytometry. Tregs were stimulated 12 h with PMA and inomycin in the present of brefeldin A. (D) Representative immunoblot and (E) quantification for 42 different cytokines and chemokines extracellularly secreted from PBS^+^ and Aβ^+^ Tregs after stimulation PMA and inomycin. (F) Tregs were labeled with anti- CD62L, CD69, CD127, PD-1, GITR, CTLA4, IL-10, Ki-67, and Helios antibodies and analyzed by flow cytometry. Data are presented as the mean ± SEM. n = 3-5; *P < 0.05, **P < 0.01.

Our previous works developed a therapeutic strategy based on Aβ_1-42_ immunization of 3xTg-AD mice 4. We expanded on prior reports to generate stable Aβ-specific Tregs *in vitro* as a promising viable immunotherapy for AD. Aβ was applied both *in vivo* and *in vitro* to generate specific Treg cells against an AD antigen for transfer into AD recipients (Figure 1A).

*In vivo* Aβ stimulation was performed in DEREG mice, which express diphtheria toxin (DT) receptor and eGFP under the control of the Foxp3 locus 8. We induced transient depletion of Tregs in naïve DEREG mice with DT treatment based on prior reports 9, 10. Then, we prestimulated DT-treated DEREG mice by immunizing them with Aβ_1-42_/CFA in addition to bvPLA2 to induce rapid rebound of Tregs against Aβ in lymphoid tissues *in vivo*. The second stimulation was carried out for 4 days *in vitro*, with splenocytes from immunized DEREG mice incubated with Aβ and bvPLA2 in the presence of anti-CD3/CD28 antibodies. Subsequent magnetic enrichment for splenic CD4^+^CD25^+^ Treg cells resulted in a highly pure Treg population (approximately 90% pure), as determined by flow cytometric analysis.

Treg suppression assays revealed that Aβ-specific Tregs (Aβ^+^ Tregs) exerted significantly greater suppressive activity against Aβ-specific Teffs than did Tregs stimulated with PBS or KLH, the most popular carrier and immunogenic protein 11 (Figure 1B). To characterize Aβ^+^ Tregs clones *in vitro*, we performed flow cytometry analysis for intracellular cytokines expression. As shown in Figure 1C, we observed that activated Aβ^+^ Tregs expressed a significant decrease in pro-inflammatory cytokines, such as interferon gamma (IFNγ), compared with that of Tregs stimulated with PBS (PBS^+^ Tregs). However, we detected a comparatively low level of pro-inflammatory cytokines, such as interleukin-17 (IL-17). Moreover, the level of transcription factor, such as T-box expressed in T cells (Tbet) and RAR-related orphan receptor gamma (RORγ) did not show significant differential expression. We further confirmed these cellular phenotypes of Aβ^+^ Tregs by assessing extracellular cytokine release with immunoblot staining analysis (Figure 1D and 1E). Overall, there was no difference in most cytokine release changes between Aβ^+^ Tregs and PBS^+^ Tregs. Interestingly, Aβ^+^ Tregs showed a significant reduction in pro-inflammatory cytokine, IL-3, while increase in anti-inflammatory cytokine, IL-10, compared with PBS^+^ Tregs. To ascertain the phenotypic characteristics of Aβ^+^ Tregs, we further assessed the levels of canonical T-cell markers cytometrically (Figure 1F). Compared to PBS-exposed Tregs, Aβ-specific Tregs showed increases in genes correlated with the activation of Tregs with suppressive potency, such as CD62L 12 and Helios 13. However, markers of CD4^+^ T-cell memory, such as CD69 14, were lower in Aβ^+^ Tregs than in PBS-exposed Tregs. Indeed, CD127 expression was not significantly different between PBS- and Aβ^+^ Tregs. Notably, Tregs stimulated *in vitro* with PBS or Aβ displayed comparable low levels (< 1%) of CD127, suggesting that *in vitro* Tregs maintained their phenotypes indicative of suppressive function 15. Together, these data suggested that *in vitro* Tregs remained stable and robust in response to Aβ peptide stimulation.

### Adoptive transfer of Aβ-stimulated Tregs restores cognitive deficits in a mouse model of Alzheimer's disease

**Figure 2:**
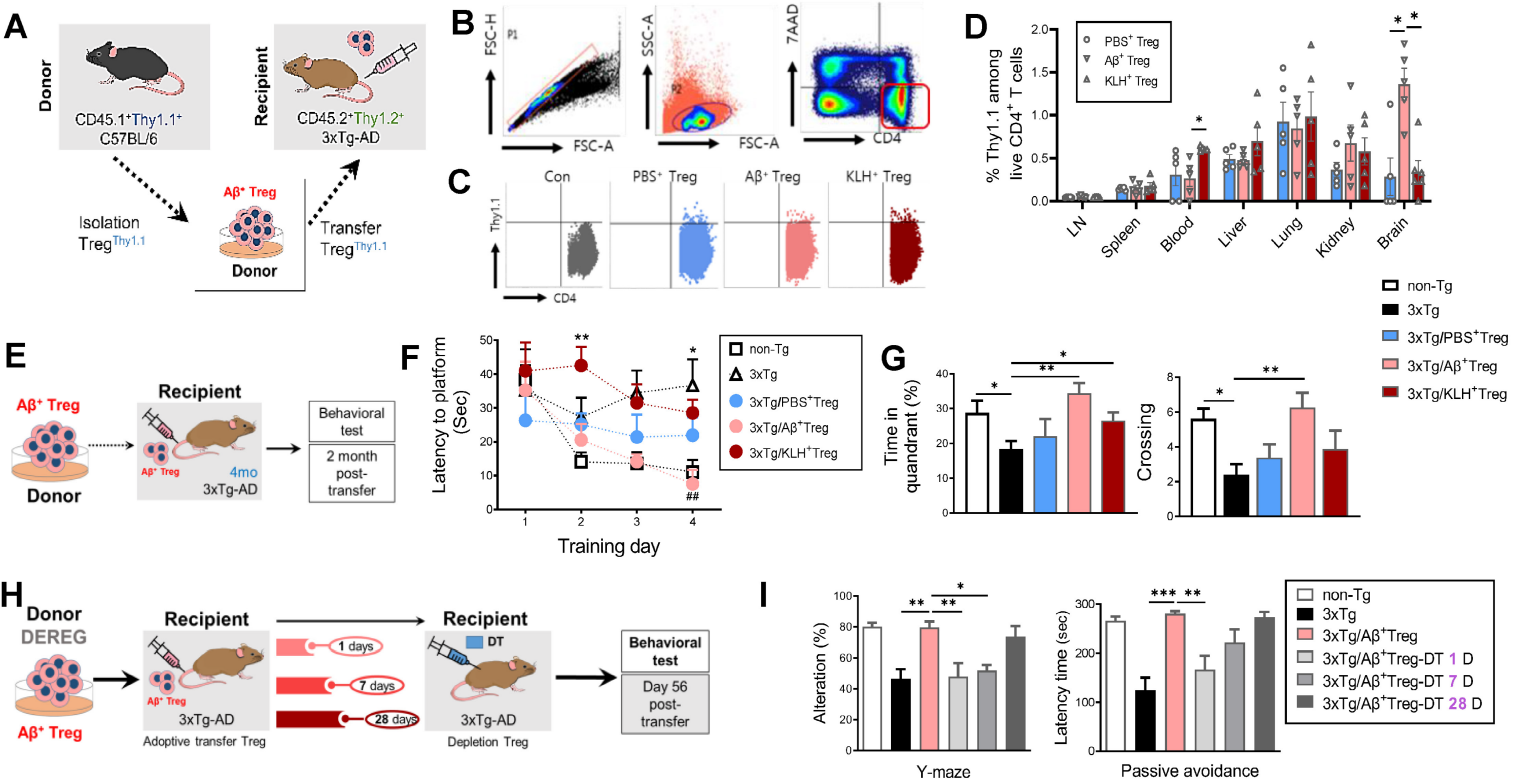
** Adoptive transfer of Aβ-stimulated Tregs rescues cognitive deficits in a mouse model of Alzheimer's disease** (A) Experimental scheme for assessing the CD45.1^+^Thy1.1^+^ Treg distribution in CD45.2^+^Thy1.2^+^ 3xTg-AD mice. (B) After 7 days, cells were harvested in several tissues and analyzed by flow cytometry. (C) Thy1.1^+^ cells were detected in CD4^+^7AAD^-^ cells. (D) The percentage of Thy1.1^+^ cells was measured in inguinal lymph nodes, spleen, blood, liver, lungs, kidneys, and brain. (E) Experimental scheme for Aβ^+^ Treg adoptive transfer into 3xTg-AD mice (recipients). (F) After two months, recipient mice were subjected to the Morris water maze test. (G) The time spent in the target quadrant and the number of platform crossings were measured. (H) Experimental scheme for transferred Treg depletion to confirm the therapeutic benefit of infused Tregs in recipient mice. (I) DT-injected recipient mice were evaluated with the Y-maze and passive avoidance tests. Data are presented as the mean ± SEM. n = 5; * P < 0.05, ** P < 0.01, *** P < 0.001.

Next, we tested whether *in vitro*-stimulated Aβ^+^ Tregs survived and were distributed following adoptive transfer. To track infused Tregs *in vivo*, we isolated Tregs from CD45.1^+^Thy1.1^+^ C57BL/6 mice and injected them into recipient CD45.2^+^Thy1.2^+^ 3xTg-AD mice via the tail vein (Figure 2A). Flow cytometric analysis on Day 7 after infusion demonstrated distinct engraftment of Thy1.1^+^ donor Tregs in a tissue-specific fashion (Figure 2B-2D). Interestingly, the relative proportion of Aβ^+^ Tregs was highest in the brain of recipient AD mice compared to other organs. These results suggest the possibility that pathogenic Aβ in AD recipients may, directly or indirectly, affect the infiltration of donor Aβ^+^ Tregs into the brain.

Over the past decade, a variety of immunization strategies have been developed by our group and others to control AD by targeting Aβ-directed immunomodulation 4, 16. Given the *in vivo* properties of *in vitro* Aβ-stimulated Tregs, we tested whether the adoptive transfer of Aβ^+^ Tregs could induce a therapeutic effect in AD by rescuing memory impairments in this disease (Figure 2E). As expected, 3xTg-AD mice displayed cognitive deficits, as demonstrated by the Morris water maze test, at 6 months of age compared to non-transgenic (non-Tg) controls (Figure 2F and 2G). Notably, Aβ^+^ Treg infusion resulted in the suppression of cognitive impairments in AD mice. This impact of transferred Tregs was not apparent in 3xTg-AD mice infused with Tregs stimulated with PBS or KLH. Additionally, we examined behavior effects of Aβ^+^ Tregs in 3xTg-AD mice at late stages of the disease with Morris water maze (MWM) test (Supplementary Figure 1). 3xTg-AD mice that received Aβ^+^ Tregs at 9 months of age exhibited increased number of entities and time in platform compared to 3xTg-AD mice but were not significantly different.

We next evaluated whether this therapeutic benefit in AD achieved with infusion of Aβ^+^ Tregs was dependent on the infused Treg cells. To answer this question, we depleted Tregs in 3xTg-AD mice with DT administered at different time points after adoptively transferring Aβ^+^ Tregs (Figure 2H). The adoptive transfer of Aβ^+^ Tregs into 3xTg-AD mice resulted in clear reductions in the impairments in spatial recognition performance measured with the Y maze test and cognitive performance measured with the passive avoidance test (Figure 2I). However, when DT was injected 1 day or 7 days after adoptive transfer, the improvements observed in Aβ^+^ Tregs-infused AD mice were lost. DT injection at later time points, such as Day 28 after Aβ^+^ Tregs adoptive transfer, still allowed effective rescue of the behavioral deficits in AD mice. These results imply that infused Aβ-specific Tregs need to be viable for longer than 7 days in mice to lead to improved AD disease outcomes.

### Aβ-stimulated Treg transfer reduces both Aβ deposition and tau phosphorylation in AD model mice

**Figure 3:**
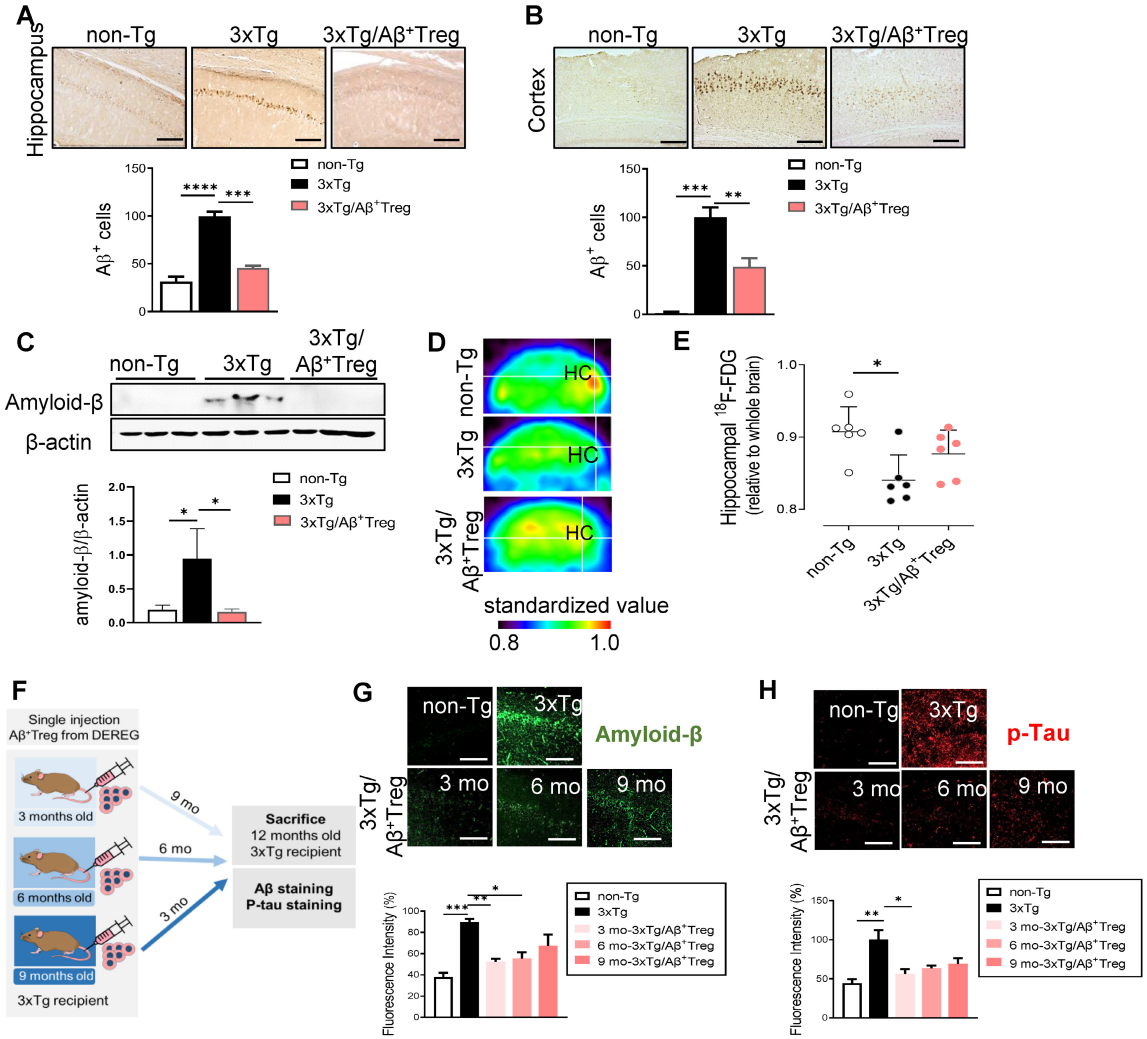
** Aβ-stimulated Treg transfer reduces both Aβ deposition and tau phosphorylation in AD model mice.** After behavioral tests were performed, mouse brains were prepared for immunohistochemistry. (A) Aβ peptides were detected in the hippocampus and (B) cortex of 3xTg-AD mice. The scale bar represents 500 μm. (C) Proteins were extracted from the hippocampus, and the levels of Aβ were measured using western blotting. (D and E) Glucose metabolism was measured using the level of hippocampal 18F-FDG determined with a PET scan. (F) Experimental scheme for assessing Aβ^+^ Treg properties in different disease stages in 3xTg-AD mice. (G) Mouse brain sections were labeled for Aβ (scale bar represents 100 μm), and the staining intensity was measured. (H) Phosphorylated tau peptides were also detected in the hippocampus (scale bar represents 100 μm), and the staining intensity was measured. Data are presented as the mean ± SEM. n = 5-6; *P < 0.05, **P < 0.01, *** P < 0.001, **** P < 0.0001.

We next assessed the role of adoptively transferred Aβ^+^ Tregs in AD by measuring Aβ levels in the hippocampus and cortex. Accumulated amyloid plaques in the hippocampus of AD mice were dramatically reduced upon adoptive transfer of Aβ-specific Tregs, as demonstrated by immunohistochemistry (Figure 3A and 3B) and western blot analysis of the hippocampal tissues of mice (Figure 3C). We further assessed cerebral glucose metabolism using ^18^F-fluorodeoxyglucose-positron emission tomography (FDG-PET), an imaging approach for AD biological definition 17. As expected, 3xTg-AD mice showed decreased ^18^F-FDG uptake in the hippocampus compared with non-Tg mice (Figure 3D and 3E). However, AD mice adoptively transferred with Aβ^+^ Tregs showed no significant alterations in brain glucose metabolism compared to WT mice. All these data may reflect the potential impact of Aβ-specific Tregs on Aβ aggregate-mediated cerebral toxicity during AD. Here, we evaluated the *in vivo* properties of Aβ^+^ Tregs in different stages of AD in 3xTg-AD mice. We intravenously infused Aβ^+^ Tregs into 3xTg-AD mice at different time points (3, 6, and 9 months of age) and sacrificed the mice at 12 months of age (Figure 3F). 3xTg-AD mice transferred with Aβ^+^ Tregs when as young as 3 months of age displayed significant reductions in the levels of Aβ aggregates (Figure 3G) and phosphorylated tau (Figure 3H) compared with those in 3xTg-AD mice. Aβ^+^ Treg transfer during the early stage of AD (3 months of age) showed the highest reduction in Aβ aggregates but transfer into AD mice at a middle age (6 months) but not those at a late age (9 months) also produced a significant decline. In particular, Aβ^+^ Tregs suppressed the levels of phosphorylated tau in the AD mice treated during the early stage of AD. Additionally, we confirmed these specific impacts of Tregs on amyloid deposition and Tau phosphorylation from DEREG mice after DT injection to induce transient depletion of Treg (Supplementary Figure 2). Altogether, these data suggest that intravenously infused Aβ^+^ Tregs may be therapeutically involved in the production and deposition of Aβ during the development of AD.

### Aβ-specific Tregs suppress neuroinflammation in microglia in 3xTg-AD mice

**Figure 4:**
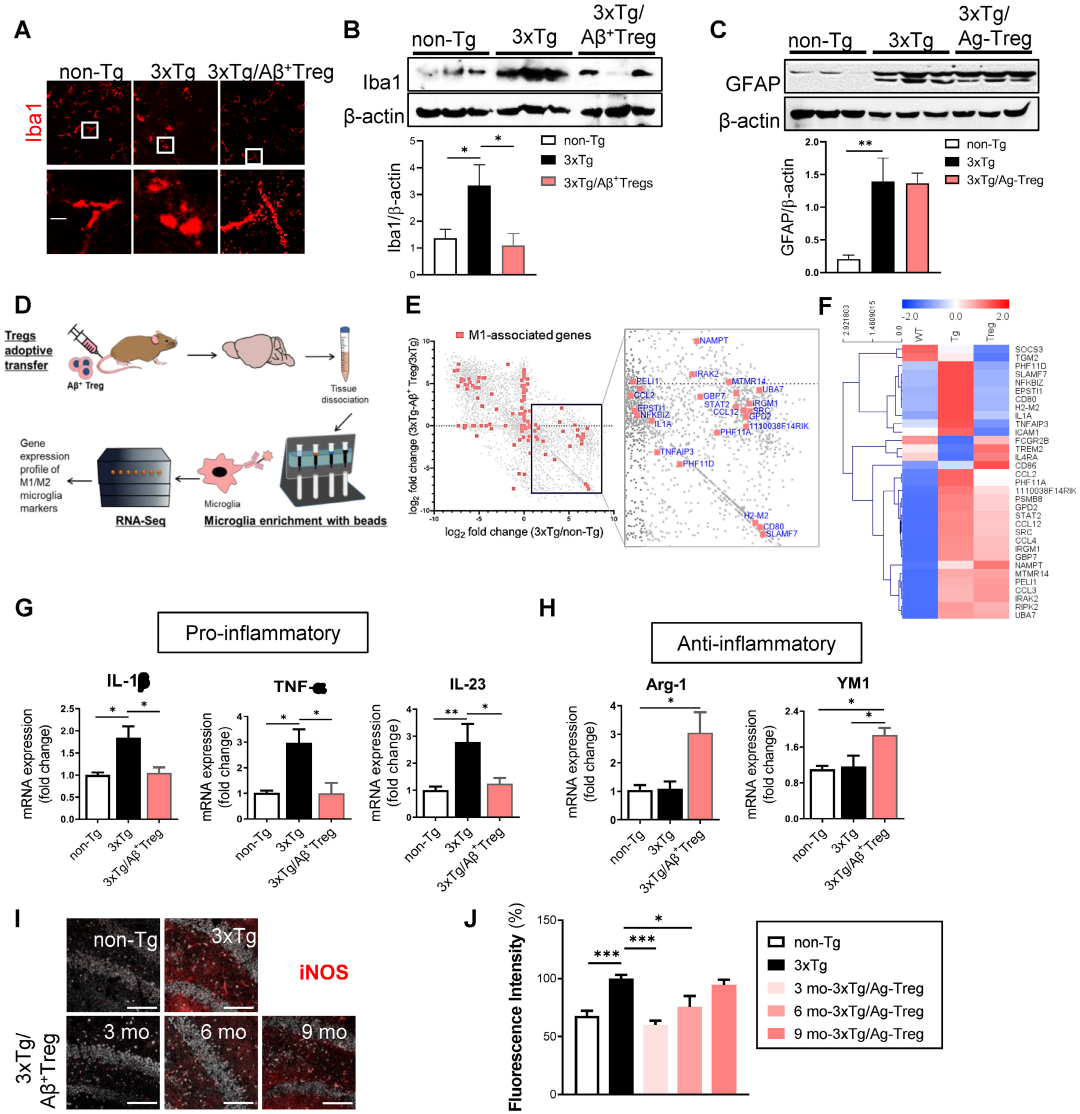
** Aβ-specific Tregs suppress neuroinflammation in the microglia of 3xTg-AD mice.** (A) Brain sections from 3xTg-AD mice were stained for Iba1 to detect activated microglia. The boxed areas indicate magnified areas. The scale bar represents 20 μm. (B) The levels of Iba1 and (C) GFAP in the hippocampus of 3xTg-AD mice were measured by western blotting. (D) Adult microglia were isolated using CD11b microbeads from the brain and mRNA was extracted for Quantseq 3`mRNA-seq. (E) RNAseq scatter plot showing M1-associated genes. (F) Heatmap of 33 microglia-associated genes differentially regulated in the Non-Tg versus 3xTg-AD mice. (G) The mRNA expression of the proinflammatory cytokines IL-1β, TNF-α, and IL-23 and (H) the anti-inflammatory cytokines Arg-1 and YM1 in the hippocampus of 3xTg-AD mice was also measured by RT-PCR. (I) The expression of NOS2 was detected in the hippocampus of 3xTg-AD mice of different ages (scale bar represents 100 μm), (J) and the staining intensity was measured. Data are presented as the mean ± SEM. n = 5; * P < 0.05, ** P < 0.01, *** P < 0.001.

Neuroinflammation is present in AD 18. During neuroinflammation, microglial activation is a unique feature observed in both AD patients 19 and animal models of AD 20. Thus, we assessed whether the adoptive transfer of Aβ^+^ Tregs influences the microglial response during AD. We first evaluated the overall microgliosis in the hippocampus through assessment of ionized calcium binding adaptor molecule-1 (Iba-1). We found activated microglia (characterized by a decreased process length and an enlarged soma area) in 3xTg-AD mice compared with non-Tg littermates (Figure 4A). Adoptive transfer of Aβ^+^ Tregs clearly suppressed reactive microglia in AD mice. The level of Iba-1 was significantly increased in AD mice compared with non-Tg mice, while Aβ^+^ Tregs clearly decreased Iba-1 upregulation in AD mice (Figure 4B).

We further examine the expression levels of glial fibrillary acidic protein (GFAP) which is widely used as a proxy of reactive astrogliosis in AD 21. Western blotting revealed the upregulated expression of GFAP in the hippocampus of 3xTg-AD mice compared with those of non-Tg mice (Figure 4C). However, no significant difference in GFAP levels was detected between 3xTg-AD mice and Aβ^+^ Tregs infused 3xTg-AD mice. Additionally, we could detect significantly decreased in the levels of myelin basic protein (MBP), an essential myelin component, in the hippocampus of 3xTg-AD mice, suggesting myelin disruption in AD (Supplementary Figure 3A and 3B). However, Aβ^+^ Tregs inhibited a reduction in MBP protein levels in 3xTg-AD mice.

Next, we performed RNA sequencing (RNA-Seq) of microglia to explore differential gene expression affected by Aβ^+^ Tregs in 3xTg-AD mice. RNA-Seq was carried out from microglia isolated from the brain samples of AD mice (Figure 4D). Analysis of gene expression profiles revealed that 5,573 genes out of final set of 23,283 were differentially expressed in 3xTg-AD mice versus non-Tg mice (Figure 4E). Interestingly, 6,293 were identified as differentially expressed in 3xTg-AD mice by adoptive transfer of Aβ^+^ Tregs. In particular, many pro-inflammatory M1-associated genes, including CCL2 and CD80, were reduced in 3xTg-AD/ Aβ^+^ Tregs mice compared to those of 3xTg-AD mice. Looking at a M1-assocated genes set (142 genes; Method), we detected that 30 genes (21.1%) were upregulated in AD mice. Surprisingly, 26 genes (86.7%) were downregulated compared to the 30 genes upregulated in the microglia of AD mice (Figure 4F).

To gain greater insight into microglial activation, the expression of pro-inflammatory cytokines, such as interleukin-1β (IL-1β), tumor necrosis factor-α (TNF-α), and interleukin-23 (IL-23) (Figure 4G), and anti-inflammatory molecules, such as Arginase-1 (Arg-1) and chitinase 3-like-3 (YM-1/CHI3l3) (Figure 4H), was quantified in the hippocampus. 3xTg-AD mice showed significantly increased mRNA levels of pro-inflammatory cytokines, while adoptive transfer of Aβ^+^ Tregs clearly decreased the upregulation of these pro-inflammatory molecules.

Indeed, late-stage 3xTg-AD mice (12 months old) displayed no significant change in pro-inflammatory cytokines, such as TNF-α and IL-1β, compared with the age-matched non-Tg mice (Supplementary Figure 3C). Aβ^+^ Tregs specifically induced a reduction of IL-1β in AD mice which were received Tregs at later stages (9 months old) under amyloid enriched environment. Similar to those of early-stage mice (Figure 4H), we also observed a significant increase in the protein levels of anti-inflammatory cytokine YM1, not Arg-1, in late-stage 3xTg-AD mice infused with Aβ^+^ Tregs (Supplementary Figure 3D). Additionally, a classic marker of reactive microglia, inducible nitric oxide synthase (iNOS) 22, was upregulated in the hippocampus of 3xTg-AD mice compared to that of non-Tg mice (Figure 4I and 4J). We found that adoptively transferring Aβ^+^ Tregs significantly decreased iNOS upregulation in AD mice. Thus, Aβ^+^ Tregs suppressed inflammation in AD, partially via regulating microglial inflammatory responses.

### Aβ-specific Tregs suppress Aβ-induced microglial inflammation through bystander suppression

**Figure 5:**
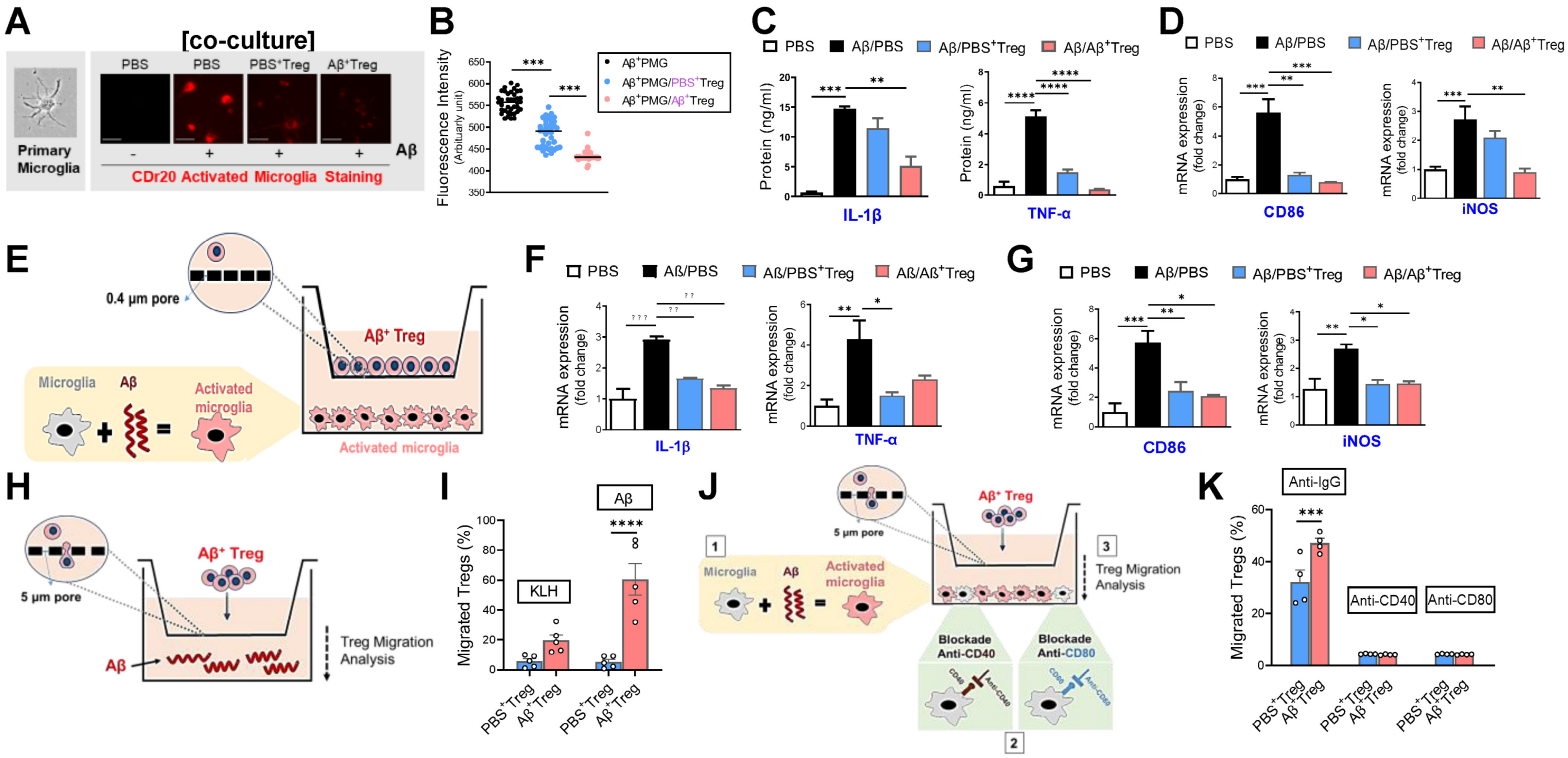
** Aβ-specific Tregs suppress Aβ-induced microglial inflammation via the bystander effect.** (A) Representative images show the primary microglial activity after 30 min of Aβ (5 μM) treatment in the presence of CDr20. The scale bar represents 100 μm. (B) Fluorescence intensity of fifty randomly selected cocultured microglia and Treg cells after 30 min of Aβ treatment with CDr20 in the red fluorescent channel (excitation: 570 nm, emission: 600 nm). (C) The secreted levels of TNF-α and IL-1β in the supernatants of cocultured cells treated with Aβ for 6 h were measured by ELISA. (D) The relative mRNA expression levels of iNOS and CD86 in cocultured microglia and Treg cells were quantified. (E) Schematic diagram for the experiment using indirect coculture with a Transwell insert (0.4 μm) to confirm the inhibitory effect of Aβ^+^ Tregs. The relative mRNA expression levels of (F) the proinflammatory cytokines IL-1β and TNF-α and (G) the microglial activation markers CD86 and iNOS were quantified in activated microglia after 6 hrs of coculture. (H) Schematic diagram for the Aβ^+^ Treg migration assay. (I) The bar graph shows the percentage of Tregs that migrated toward Aβ compared to KLH. (J) Schematic diagram of the migration assay performed with Aβ^+^ Tregs and anti-CD40 or anti-CD80 antibody-treated primary microglia. (K) The bar graph shows the percentage of Tregs that migrated toward antibody-treated microglia. Data are presented as the mean ± SEM. n = 3-4 per group; * P < 0.05, ** P < 0.01, *** P < 0.001, **** P < 0.0001.

Within the CNS, microglia can interact with infiltrated antigen-specific T cells during neurodegeneration through direct contact or bystander suppression 23. Given the *in vivo* properties of adoptively transferred Aβ^+^ Tregs, we asked whether Aβ-specific Treg cells directly impact the microglial phenotype in response to the Aβ antigen. First, we monitored the inflammatory response of primary microglia by performing a real-time imaging analysis using the microglia-specific high-performance fluorogenic chemical probe CDr20 24 (Figure 5A and 5B). Live-cell imaging with CDr20 revealed that exposure to Aβ for 6 h induced an increased CDr20 signal, indicating enhanced induction of microglial activation by Aβ. However, coculture with Aβ-specific Tregs effectively inhibited inflammation in primary microglia induced by Aβ exposure, as measured with the CDr20 imaging analysis.

Consistent with previous reports 25, we observed enhanced levels of the pro-inflammatory cytokines IL-1β and TNF-α in primary microglia stimulated with Aβ (Figure 5C). Furthermore, microglial activation markers such as iNOS and CD86 were increased in Aβ-treated primary microglia (Figure 5D and Supplementary Figure 5). Interestingly, such Aβ-induced microglial inflammation was effectively suppressed by coculture with Aβ-specific Tregs, suggesting the ability of Aβ^+^ Tregs to alter the inflammatory phenotype of microglia induced by Aβ. Next, we asked whether this inhibitory effect of Aβ^+^ Tregs was achieved by direct contact with microglia. To answer this question, we performed Transwell analysis using an exceptionally small Transwell pore size (i.e., 0.4 µm) to allow transport of only small chemical compounds (Figure 5E). Similar to the results from the coculture system, we observed an inhibitory role for Aβ^+^ Tregs in microglial inflammation when microglia activated with Aβ were seeded in the lower chamber and Aβ^+^ Tregs were seeded in the upper chamber; this role was supported by measurements of pro-inflammatory cytokines (Figure 5F) and microglial activation markers (Figure 5G). Therefore, these results may indicate that antigen-specific Tregs can regulate the microglial inflammatory phenotype, in part through an indirect contact-mediated mechanism.

Next, we investigated whether *in vitro* Treg cells are capable of migrating toward the Aβ antigen by performing a Transwell (pore size: 5 µm) migration assay with Tregs and the Aβ antigen (Figure 5H). Surprisingly, when Aβ was added to the medium, Treg cells significantly migrated toward the Aβ antigen compared to KLH (Figure 5I). In particular, Aβ-specific Tregs showed the highest level of migration toward the Aβ antigen following Aβ addition. Additionally, we further evaluated the possibility that Aβ^+^ Tregs migrate toward activated microglia in a manner induced by Aβ. As shown in Figure 5K, Aβ-activated microglia were seeded in the lower chamber, and then Aβ^+^ Tregs were seeded in the upper chamber. Microglia activated with Aβ potently induced Aβ^+^ Treg migration toward the microglia compared with that of PBS^+^ Tregs. However, this migratory effect on Aβ^+^ Tregs was clearly blunted when anti-CD40 or anti-CD80 antibody-treated primary microglia were used. These results may indicate bystander suppression in which antigen-specific Tregs promote immune tolerance by secreting cytokines to modulate immune responses during neurodegeneration.

## Discussion

Since the first clinical trial of adoptively transferred human Tregs was reported in 2009 26, a wide range of treatment strategies have been developed and applied 27. We propose a novel strategy to generate Aβ-specific Tregs through both *in vivo* and *in vitro* stimulation and subsequently demonstrate the therapeutic efficacy of these Treg cells in controlling neurodegenerative activities in AD. In recent decades, many studies performed by our groups and others have demonstrated that Treg induction elicits beneficial effects on neurodegenerative diseases, including PD 5, 28 and ALS 29. Indeed, our groups have already started clinical trials in AD and are currently conducting phase I clinical trials with the Treg technology described in this study 30.

As of now, adaptive immunity has been receiving interest as a new therapeutic target for AD. Dansokho et al. provided evidence that transient depletion of Treg cells accelerated the cognitive impairment in APP/PS1 transgenic mice 31. Interestingly, amplification of Tregs through peripheral IL-2 treatment restored cognitive function in APP/PS1 mice. A more recent study from AD patients revealed that the immunophenotype and suppressive function of Tregs could be enhanced by *ex vivo* expansion 32, which suggests the prospects of Treg adoptive cell transfer for AD treatment. Importantly, the success of Treg-based immunotherapies depends on multiple factors. Here, we first generated Aβ antigen-specific Tregs by both *in vitro* and *in vivo* stimulation with Aβ peptide. These Treg clones expanded *in vitro* system retain antigen specificity as demonstrated by binding with MHC-Aβ peptide tetramer in a dose-dependent manner. Aβ^+^ Tregs clones showed highly suppressive activity compared to Tregs stimulated with either PBS or keyhole limpet hemocyanin (KLH), an immunogenic protein antigen as a control antigen. Moreover, these Tregs displayed distinct cell phenotypes for their immunosuppressive function assessed by immunoblot staining of extracellular cytokine release including chemokines CCL3 (MIP-1α) and CCL4 (MIP-1β). We thus propose new therapeutic strategy to generate stable and robust Aβ antigen-specific Tregs with highly functional and phenotypic features of Tregs.

A growing body of evidence now indicates that systemic inflammation can affect the pathogenesis and progression of AD 33. It is not surprising that numerous clinical trials are testing vaccines for AD 34, suggesting a pivotal role of peripheral immune activity in AD. Surprisingly, Aβ antigen in the brain can drain to peripheral lymph nodes 35 and be then presented to T cells by APCs in the periphery 23. These events may initiate peripheral adaptive immune activation 20, 36, 37. These Aβ-derived adaptive immune responses are likely to be occurred, mainly, in the early stage of AD. As the disease progresses, meningeal lymphatic function is impaired resulting in the blockage of Aβ drain and Aβ deposition in the meninges 38. Here, we compared the therapeutic potential of Aβ^+^ Tregs adoptive transfer into 3xTg-AD mice at different stages of AD. 3xTg-AD mice displayed highly reduced amyloid accumulation as well as neuroinflammation when Aβ^+^ Tregs were adoptively transferred into mice at the early stage of AD. Surprisingly, these therapeutic impacts of single adoptive transfer Aβ^+^ Tregs could be maintained for more than 6 months *in vivo*. It therefore appears that our study and the recent reports on Tregs support a similar conclusion about the role of peripheral immune reaction in development and pathogenesis of AD.

Indeed, in the healthy brain, the entry of peripheral immunocytes is tightly controlled to maintain homeostasis. However, under neuroinflammatory conditions, immune responses inside and outside of the CNS result in the infiltration of CNS antigen-specific T cells from the peripheral lymph nodes into the brain 23, 36. In response to neuroinflammatory stimuli, microglia are capable of stimulating CD4^+^ and CD8^+^ T cells in the brain. The precise role of infiltrated CD4^+^ T cells in the CNS during neurodegeneration is partially dependent on the phenotype of the T cells 39. For instance, inflammatory Aβ-reactive effector T cells (Teffs) strongly accumulate at sites of disease pathology 40. Interestingly, Aβ-specific Th1 cells enhance microglial activation, the Aβ plaque burden, and cognitive impairments in AD mice in a manner dependent on IFN-γ. Additionally, adoptive transfer of Aβ-specific Th1 and Th17 Teffs exacerbates these outcomes related to neurodegeneration in AD mice 20, suggesting a pathological role for Aβ-specific Teffs in AD progression. Apparently, Th1 and Th17 Teffs drive proinflammatory responses, while CD4^+^CD25^+^Foxp3^+^ Tregs exert anti-inflammatory and immunosuppressive functions 36. In contrast, a decreased frequency of CD4^+^CD25^+^Foxp3^+^ Tregs was reported in patients with AD compared to age-matched controls 41. Interestingly, recent in-depth analysis of immune cells demonstrated that Tregs exist in the brain 42, 43. Although the exact function of brain Tregs are still unclear, Tregs in the brain are capable of directly suppress neuroinflammation 36, 44. However, in the chronically inflamed brain of neurodegenerative diseases, the frequency and suppressive properties of Tregs in the brain are reduced, while the frequency and function of Teffs are increased 45. It is therefore likely that the imbalance between Tregs and Teffs within the CNS directly determines disease progression in neurodegeneration, which should be addressed in future studies. Altogether, these results suggest an implication of Tregs in the pathogenesis of AD, but their actual impact on disease progression needs to be further studied.

Altered microglial phenotypes subsequently determine functional outcomes in health and disease 36. Hence, the unique microglial phenotype associated with each neurological disease specifically defines the role of microglia in a disease-specific manner. In the context of AD, the roles of microglia in the initiation and progression of AD are currently unclear and debated, with reports indicating both harmful and beneficial impacts on AD 46. A unique microglial phenotype associated with AD, disease-associated microglia (DAM), was recently identified 47. DAM appear to be differentiated through a two-step process, probably inducing different AD outcomes in a disease stage-dependent manner. Stage 1 DAM transition from the homeostatic state, which results in protective DAM against Aβ at early stages, while at late states, DAM become dysfunctional and exacerbate disease.

## Conclusion

In summary, we presented a strategy to generate robust and stable Aβ-specific Tregs by both *in vivo* and *in vitro* stimulation with Aβ. Adoptive transfer of Aβ-specific Tregs into AD mice resulted in therapeutic effects on AD, including a reversal of AD-associated cognitive decline and a reduction in Aβ accumulation. Specifically, we confirmed that Aβ-specific Tregs inhibited neuroinflammatory responses by, in particular, influencing phenotypic changes in microglia promoting deactivation. Our results reveal a close link between microglia and Treg cells, with a focus on the potential role of Tregs in microglial immunophenotypic shifts in AD. Our proof-of-concept preclinical study suggests that immunomodulatory approaches based on CNS-driven antigen Aβ-specific Tregs could be therapeutic strategies for reversing or slowing disease progression in AD.

## Supplementary Material

Supplementary materials and methods, figures.

## Abbreviations

AD): Alzheimer's disease
Aβ): amyloid-beta
Aβ^+^ Tregs): Aβ antigen-specific Tregs
Arg-1): Arginase-1
YM-1/CHI3l3): chitinase 3-like-3
DAM): disease-associated microglia
DT): diphtheria toxin
Iba-1): ionized calcium binding adaptor molecule-1
IL-1β): interleukin-1β
IL-23): interleukin-23
iNOS): inducible nitric oxide synthase
PBMCs): peripheral blood mononuclear cells
Tregs): regulatory T cells
TNF-α): tumor necrosis factor-α
