# Supplementary material for: Adoptive therapy with amyloid-β specific regulatory T cells alleviates Alzheimer's disease
Source: Theranostics. 2022 Nov 7;12(18):7668–80. doi: 10.7150/thno.75965 (PMC9706584; doi:10.7150/thno.75965)
Supplement: Supplementary file 1 — Supplementary materials and methods, figures. [file thnov12p7668s1.pdf]

Supplementary Materials

Materials and Methods

Ex vivo-expanded mouse Tregs injection

To compare the effects of Tregs according to progression of AD, Aβ^+ Tregs (1×10^6) were adoptively transferred to 3-, 6-, and 9-month-old 3xTg-AD mice, which represented early, middle, and late phase of AD, respectively. The adoptively transferred mice were euthanized at the age 12 month. Furthermore, 2×10^5, 1×10^6, or 5×10^6 Aβ^+ Tregs were adoptively transferred to confirm the dose-dependent effect of Tregs.

Flow cytometry

To determine the purity and phenotypes of isolated murine Tregs, cells were stained with antibodies specific for the following markers: CD4-PE-Cy7 (clone GK1.5; Invitrogen, Waltham, MA, USA, #25-0041-82), CD4-PE-Cy5 (clone GK1.5; Invitrogen, #15-0041-82), CD25-APC-Cy7 (clone PC61; BD Pharmingen, Franklin Lakes, NJ, USA, #557658), CD69-FITC (clone H1.2F3; BD Pharmingen, #557392), CD127-PE (clone A7R34; Invitrogen, #12-1271-82), CD62L-APC (clone MEL-14; Invitrogen, #17-0621-82),), PD-1-BB515 (clone RMP1-30; BD bioscience, #566832), CTLA4-PE (clone UC10-4B9, Invitrogen, #12-1522-82), and GITR-PE (clone DTA-1; Invitrogen, #12-5874-82). For intracellular staining, cells were fixed and permeabilized using the FoxP3/Transcription Factor Fixation/Permeabilization Kit (eBioscience, San Diego, CA, USA, #00-5521-00) for 30 min and stained with an anti-Helios-AF647 (clone 22F6; eBioscience, #51-9883-82), Ki-67-PE-Cy7 (clone SolA15; eBioscience, #25-5698-82), IL-10-APC (clone JES5-16E3; eBioscience, #17-7101-82), IFNr-FITC (clone XMG1.2; eBioscience, #11731182), IL-17a-APC-Cy7 (clone TC11-18H10.1; BioLegend, San Diego, CA, USA, #506939), anti-Tbet-eFluor 660 (clone 4B10; eBioscience , #50582582) and anti-RORγ-PerCp eFluor 710 (clone B2D; eBioscience #46698182).

To detect adoptively transferred Tregs, cells were stained with an anti-Thy1.1-FITC antibody (clone HIS51; Invitrogen, #11-0900-81), 7AAD (Invitrogen, #00-6993-50) and an anti-CD4-PE-Cy7 antibody. The stained cells were harvested and analyzed on a BD FACSLyric (BD Bioscience) flow cytometer. Data were acquired using BD FACSuite software (BD Bioscience).

mRNA expression profiling

For brain mRNA expression profiling, $A\beta^+$ Tregs (1×10^6) were adoptively transferred to 9-month-old 3xTg mice, and the brain were harvested after 14 days. After percoll density gradient centrifugation, microglia were isolated using CD11b (Microglia) Microbeads (Miltenyi). RNA was isolated using an easy-BLUE RNA extraction kit (iNtRON Biotechnology, Seongnam, Korea, #17061) and processed for QuantSeq 3' mRNA-Seq.

Intracellular and Extracellular Cytokine analysis

To induce cytokine production, PBS^+ and $A\beta^+$ Tregs were stimulated by 20 ng/ml PMA, 1uM inomycin, and 3 µg/ml brefeldin A (eBioscience) for 12 h, then staining was performed for flow cytometry. For analysis of extracellular cytokine release, PBS^+ and $A\beta^+$ Tregs were stimulated by 20 µg/ml PMA and 1uM inomycin for 12 h and culture supernatants were analyzed using proteome profiler mouse cytokine array kit (R&D Systems, Minneapolis, MN, USA, #ARY006)

Behavior tests

The spatial learning and memory of 3xTg-AD mice were examined using the Morris water maze test with minor modifications [1]. All mice were subjected to three trials per day at intervals of 15 min for four consecutive days. For the probe trial, the platform was removed from the pool, and the mice were allowed to swim freely for 60 sec to search for the previous location of the platform. The escape latency, time spent in the platform quadrant, and number of platform crossings were recorded for each mouse. Data were collected using a video camera connected to a video recorder and tracking device (S-MART; Pan-Lab, Barcelona, Spain).

To confirm the effects of DT injection and human Aβ$^+$ Treg transfer, Y-maze and passive avoidance tests were performed. Spatial working memory was examined with the Y-maze test, with the maze consisting of three identical arms at 120° from each other. Mice were placed in an arm facing the wall of the arm and allowed to freely navigate through all three arms for 5 min, and their behavior was recorded. The passive avoidance test was performed to estimate the learning and memory capacity. The apparatus consisted of a lighted chamber and a dark chamber (20 × 20 × 30 cm) divided by a door. On the training day, mice were placed in the lighted chamber facing away from the door. Once the mouse entered the dark chamber, the door was closed, and a foot shock (0.35 mA, 2 sec) was delivered. Thirty seconds after the shock, the mouse was removed. Mice were given trials for two days. On the test day, the mice were placed in the lighted chamber, and the latency to dark chamber entry was recorded.

Immunohistochemistry analysis

After the behavioral tests were performed, mice were anesthetized with isoflurane and transcardially perfused with PBS. The brain was postfixed in 4% paraformaldehyde at 4°C overnight (12-16 h), transferred to a 30% sucrose solution, frozen and then cryosectioned on a sliding microtome into 30-μm-thick coronal sections. The brain sections were heated in 10 mM sodium citrate buffer (pH 6.0) in an autoclave and further incubated for 20 min with 3% hydrogen peroxide (Sigma–Aldrich) to quench endogenous peroxidase activity. After washing with PBS, nonspecific binding was reduced by blocking the sections with 5% bovine serum albumin in PBS for 1 h. The sections were incubated with a mouse anti-Aβ monoclonal antibody (1:500; BioLegend, #800701) or anti-Myelin Basic Protein (MBP) antibody (1:500; Abcam, Cambridge, UK, #ab40390) overnight at 4°C. The brain sections were washed with PBS, incubated with a biotinylated secondary antibody, and processed with an avidin-biotin complex kit (Vectastain ABC kit; Vector Laboratories, Burlingame, CA, USA, #PK-6102 or

PK-6101) at RT. The sections were visualized by incubation with 0.05% diaminobenzidine-HCl (Vector Laboratories, #SK4100). The labeled sections were then mounted and analyzed under a bright-field microscope (Nikon, Tokyo, Japan).

For immunofluorescence, coronal sections of the hippocampus, starting rostrally from −2.1 mm anteroposterior and continuing to −4.5 mm anteroposterior relative to the bregma, were incubated overnight at 4°C with the following primary antibodies: anti-Iba1 (1:2000; Abcam, #ab5076), anti-iNos (1:500; Santa Cruz Biotechnology, Dallas, TX, USA, #sc-7271), anti-Aβ (1:500; BioLegend), and anti-p-Tau (1:500; Invitrogen, #MN1020). The sections were subsequently incubated for 2 h at RT with Alexa Fluor 488- or Alexa Fluor 594-conjugated IgG secondary antibodies and then counterstained with DAPI. The sections were examined using an LSM 800 confocal laser-scanning microscope (Carl Zeiss, Oberkochen, Germany). The staining intensity was measured and quantified using ImageJ software (http://rsb.info.nih.gov/ij/). Staining images were split with an ImageJ plug-in to obtain a monochromatic version. The channel corresponding to the stain of interest was used for measurement. The percentage of the staining intensity was calculated relative to the 3xTg group and multiplied by 100.

Western blot analysis

Cells were lysed with PRO-PREP (iNtRON Biotechnology, #17081) in ice-cold conditions (4°C) for 20 min. For insoluble Aβ detection, western blotting was performed as described previously with minor modifications [2]. Samples were extracted using 80% formic acid and then processed with a glass Dounce homogenizer. The protein concentration of each sample was determined using the Bradford assay. Samples (30 μg of protein) were resolved on a 10% sodium dodecyl sulfate-polyacrylamide gel and transferred to a nitrocellulose membrane, which was blocked for 30 min in 5% skim milk at room temperature. After 30 min of blocking,

the membrane was incubated overnight at 4°C with specific antibodies against CD86 (1:1000; Cell Signaling Technology, #91882), iNOS (1:2000; Santa Cruz Biotechnology), Iba-1 (1:2000; Abcam), GFAP (1:250; Invitrogen, #13-0300) or β-actin (1:5000; Santa Cruz Biotechnology, #sc1616). After washing with Tris-buffered saline containing 25 mM Tris-Cl, 150 mM NaCl, and 0.05% Tween-20, the membrane was incubated for 2 h with a horseradish peroxidase-conjugated secondary antibody (anti-rabbit or anti-mouse IgG). Immunoreactivity was visualized with a Western Blotting Detection Reagent Kit (Thermo, #PI32106). Antibody binding was determined using a chemiluminescence detection system. Densitometric analysis was performed using ImageJ.

RNA extraction and RT–qPCR assays

Total RNA was isolated from brain tissue using an easy-BLUE RNA extraction kit, and cDNA was synthesized using Cyclescript reverse transcriptase (Bioneer, Deajeon, Korea, #E-3131). The samples were prepared for RT–qPCR using a SensiFAST SYBR no-Rox kit (Bioline, London, UK, #BIO-98020). The cycling conditions were 95 °C for 10 s, 55 °C for 10 s, and 72 °C for 10 s, and RT–qPCR was performed using a CFX Connect System (Bio–Rad, Hercules, CA, USA). The expression of target mRNAs was analyzed by the ΔΔCt method, and mouse β-actin was used as an endogenous control. All fold changes were calculated relative to the control group. The base sequences of the primers were as follows: *Cd86*: forward, 5′–GACCGTTGTGTGTGTTCTGG–3′ and reverse, 5′–GATGAGCAGCATCACAAGGA–3′; *iNOS*: forward, 5′–CAGCTGGGCTGTACAAACCTT–3′ and reverse, 5′–CATTGGAAGTGAAGCGTTTCG–3′; *TNF-α*: forward, 5′–GGCAGGTTCTGTCCCTTTCAC–3′ and reverse, 5′–TTCTGTGCTCATGGTGTCTTTTCT–3′; *Il-23*: forward, 5′–CCTTCTCCGTTCCAAGATCCT–3′ and reverse, 5′–

ACTAAGGGCTCAGTCAGAGTTGCT–3′; *Il-1β*: forward, 5′–AAGCCTCGTGCTGTCGGACC–3′ and reverse, 5′–TGAGGCCCAAGGCCACAGG–3′; *Ym1*: forward, 5′–TGGAGGATGGAAGTTTGGAC–3′ and reverse, 5′–GAGTAGCAGCCTTGGAATGT–3′; *Arg-1*: forward, 5′–CTCCAAGCCAAAGTCCTTAGAG–3′ and reverse, 5′–AGGAGCTGTCATTAGGGACATC–3′; and *β-Actin*: forward, 5′–GTGCTATGTTGCTCTAGACTTCG-3′ and reverse, 5′–ATGCCACAGGATTCCATACC–3′.

ELISA

To measure IL-1β and TNF-α in conditioned media from cocultures of primary microglia and PBS^+ Tregs or $A\beta^+$ Tregs, coculture supernatants were collected after polarization with Aβ (6 h) and analyzed by ELISA according to the manufacturer's protocol (R&D Systems, #DY401 and #DY410).

Supplementary Figures

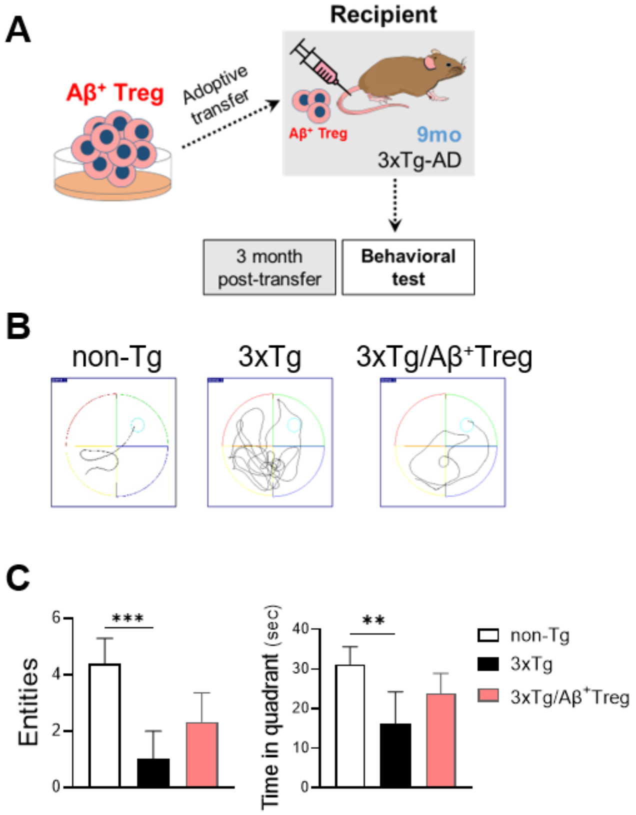

Supplementary Figure S1. (A) Experimental scheme for $A\beta^{+}$ Treg adoptive transfer into 9-month-old 3xTg-AD mice. (B) After three months, recipient mice were subjected to the Morris water maze test. (G) The number of platform entries and the time spent in the target quadrant were measured. Data are presented as the mean ± SEM. n = 5-6 per group; ** $P < 0.01$, *** $P < 0.001$.

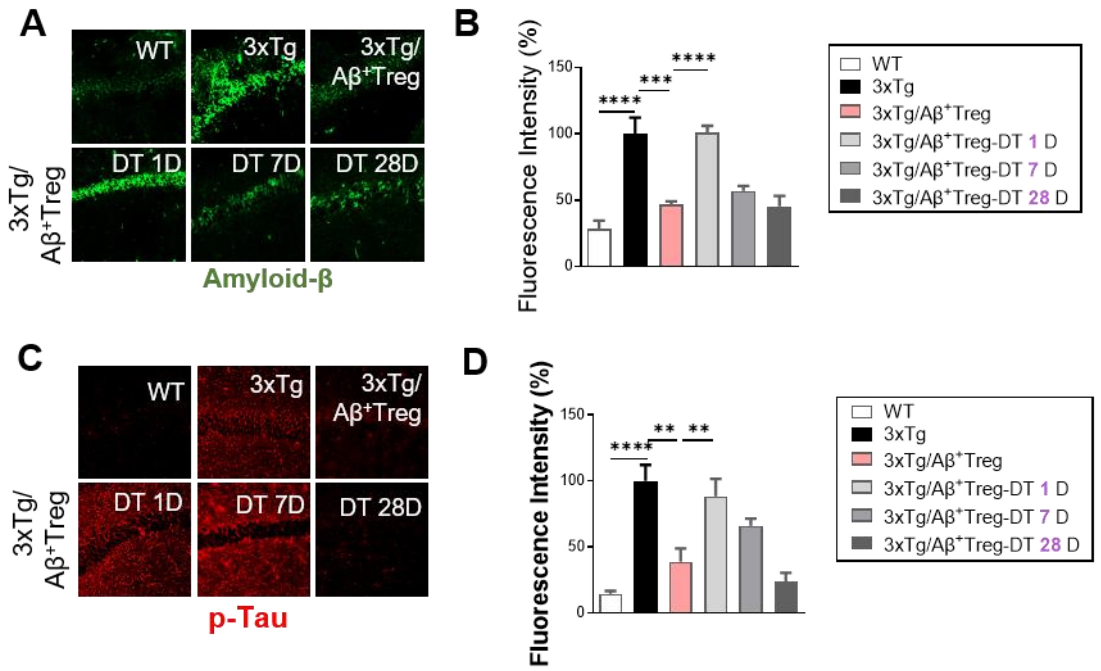

Supplementary Figure S2. Expression of Aβ and phosphorylated Tau in the hippocampus of recipient 3xTg-AD mouse brains after DT treatment. (A) Aβ peptides were detected in the hippocampus by immunostaining, and (B) the staining intensity was measured. (C) The expression of phosphorylated tau was detected in the hippocampus, and (D) the staining intensity was measured. Data are presented as the mean ± SEM. ** P <0.01, *** P <0.001, **** P <0.0001.

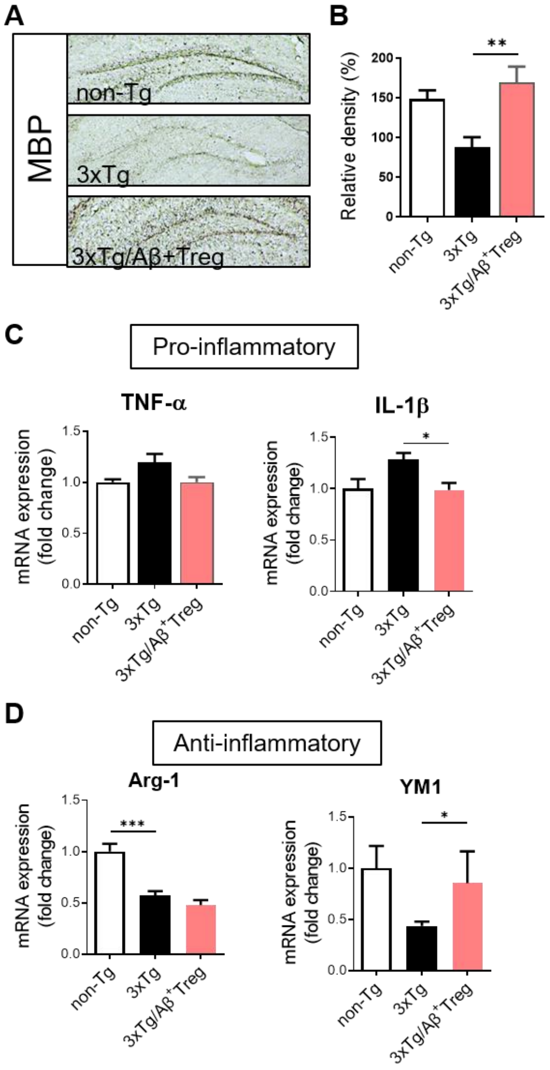

Supplementary Figure 3. (A) MBP were detected in the hippocampus of 3xTg-AD mice. (B) The relative density was measured. (C) The mRNA expression of the proinflammatory cytokines TNF-α and IL-1β and (D) the anti-inflammatory cytokines Arg-1 and YM1 in the hippocampus of 9-month-old 3xTg-AD mice was also measured by RT–PCR. Data are presented as the mean ± SEM. n = 3-6; *P<0.05, ** P <0.01, *** P <0.001.

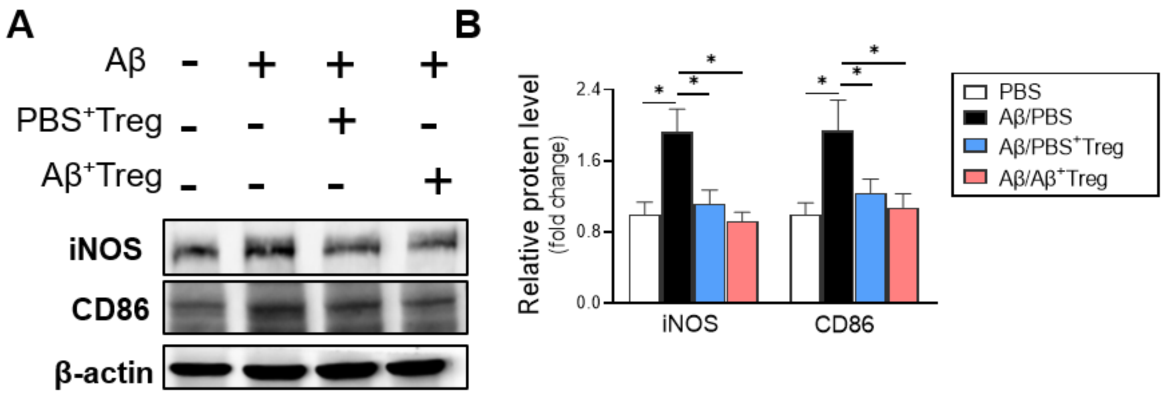

Supplementary Figure 4. Protein expression of iNOS and CD86.

(A) Immunoblot analysis of iNOS, CD86, and β-actin in cocultured microglia and Treg cells.

(B) Bar graph showing the relative protein levels of iNOS and CD86. Data are presented as the mean ± SEM. * P <0.05.

References

1. Kim HY, Lee DK, Chung BR, Kim HV, Kim Y. Intracerebroventricular Injection of Amyloid-beta Peptides in Normal Mice to Acutely Induce Alzheimer-like Cognitive Deficits. J Vis Exp. 2016.

2. Klunk WE, Pettegrew JW. Alzheimer's beta-amyloid protein is covalently modified when dissolved in formic acid. J Neurochem. 1990; 54: 2050-6.
